# Acute calcific epicondylitis associated with primary hypoparathyroidism: a paradox effect or an adverse event

**DOI:** 10.1093/rap/rkaa007

**Published:** 2020-03-10

**Authors:** Basem Awadh, Abdul Wahab Al-Allaf

**Affiliations:** Rheumatology Division, Department of Medicine, Hamad General Hospital, Doha, Qatar

**Key message**

- Hypoparathyroidism could induce calcific epicondylitis in young people. We highlight the pathophysiology of calcific tendonitis.

Sir, Calcific tendonitis is characterized by the accumulation of basic calcium phosphate hydroxyapatite crystals within the tendon. The supraspinatus tendon is the most commonly affected [1]. Calcific epicondylitis has rarely been reported as a cause of acute elbow pain [2]. Calcific tendonitis is mainly idiopathic, but it can be associated with other diseases [3, 4]. We describe a previously unreported case of calcific epicondylitis in a patient with primary hypoparathyroidism on a high dose of calcium supplement, with a literature review for calcific tendonitis and its proposed pathogenesis.

**Fig. 1:**
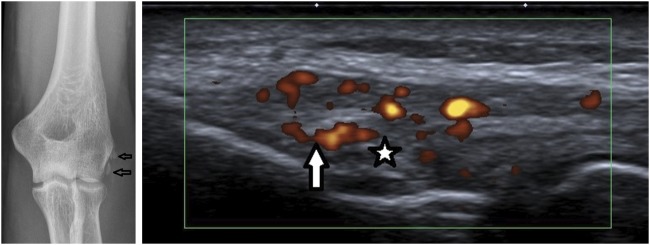
Left elbow X-ray and US: calcification and hyperaemia at the common extensor tendon Anteroposterior X-ray of the left elbow shows two amorphous calcifications (arrows) at the lateral epicondyle of the left humerus. US image of the common extensor tendon and the calcification (star) and hyperemia (arrow) on colour Doppler around the calcification

A 33-year-old man presented with acute left elbow pain and swelling. His elbow X-ray showed two hyperdense calcifications at the lateral epicondyle. The US scan revealed localized hyperechoic deposits over the lateral epicondyle with significantly increased Doppler activity, confirming the clinical diagnosis of acute lateral calcific epicondylitis (Fig. 1). The work-up for inflammatory arthritis was unremarkable. He has been treated with celecoxib PO 200 mg daily for 5 days, with a good response. Immediately before that, he complained of a foreign body sensation while swallowing, and CT of the neck revealed a tiny calcific hyperdensity along the posterior surface of the soft palate adjacent to the left vallecula.

Upon reviewing his file, he was found to have had primary hypoparathyroidism since 2015. Before this diagnosis, he was seen a few times because of non-specific symptoms of irritability, generalized weakness and numbness. His investigation revealed hypocalcaemia of <1.5 mmol/l, with a low PTH of 3 pg/ml (normal is 15–65 pg/ml) and unremarkable ECG. His serum calcium was corrected to the lower normal level, which required a high daily intake of calcium carbonate of 1250 mg (500 mg calcium), three tablets three times daily, and calcitriol 1 μg/day. His 24-h urinary calcium was high at 11.8 mmol/24 h (normal range is 2.5–7.5 mmol/24 h). He has been referred to an endocrinologist to consider PTH replacement therapy to optimize his management.

Calcific tendonitis is largely idiopathic and can be associated with trauma and tissue hypoxia in up to one-third of the patients [3]. There are two proposed mechanisms for calcific tendonitis: degenerative and reactive [4]. The degenerative calcification theory proposes that dystrophic calcification of the tendon follows a necrotic phase, secondary to wear and tear attributable to ageing. This is supported by the observation that calcific tendonitis seldom affects people before the fourth decade [5]. In contrast, the reactive calcification theory involves four phases (pre-calcific, formative, resorptive and healing) and is supported by a variety of imaging studies demonstrating a complete resolution of the calcium deposits [4, 5].

However, calcific tendonitis has been reported in association with hypothyroidism, type I diabetes mellitus and hyperparathyroidism [1, 6]. Hypoparathyroidism has been reported as a cause of rotator cuff tendonitis, but not in the epicondyle area as in our case [7].

In our case, given the absence of trauma or any other predisposing factors and given his young age, the calcific tendonitis is most likely to be secondary to the underlying hypoparathyroidism. However, it is not clear whether his calcific tendonitis is attributable to his primary disease or to the high level of calcium replacement, which was evidenced by hypercalciuria. Calcium–phosphorus imbalance (low calcium, 1.32 mmol/l, and high phosphorous, 1.95 mmol/l) or hyperphosphataemia is the proposed mechanism involved. High-calcium supplements are also known to cause soft tissue calcifications (cardiovascular and kidneys). It is well known that primary hypoparathyroidism is associated with calcification of the basal ganglia both before and after initiation of proper treatment [8, 9].

A better understanding of the pathophysiology of calcific tendonitis is vital for the prevention and management of this condition. In young patients with calcific tendonitis, hypoparathyroidism should be considered as a causative factor.

*Funding:* No specific funding was received from any funding bodies in the public, commercial or not-for-profit sectors to carry out the work described in this manuscript.

*Disclosure statement:* The authors have declared no conflicts of interest.
